# Intra-amniotic and systemic administration of methotrexate with concomitant surgical evacuation of 11 + 5 weeks cervical ectopic pregnancy: a case report

**DOI:** 10.1186/s12884-023-05794-0

**Published:** 2023-07-01

**Authors:** Ahmed Sameh Moustafa Ghoubara, Juhayna Samier Ahmed Elsheikh, Hossam Ramadan Abdulwahab, Ahmed Abdelrahem Ahmed Taha

**Affiliations:** Obstetrics and Gynecology, Aswan University Hospitals, Aswan, 81511 Egypt

**Keywords:** Cervical pregnancy, Ectopic pregnancy, Methotrexate

## Abstract

**Background:**

Cervical pregnancy is a rare type of ectopic pregnancy. The management of cervical pregnancy is challenging because of the rarity of the condition, late presentation, which is associated with increased risk of failed medical treatment, and excessive post-evacuation bleeding that may require hysterectomy. There is no good evidence in the literature regarding the pharmacological management of living cervical ectopic pregnancy of more than 9 + 0 weeks of gestation, and there is no standard protocol on methotrexate doses in these cases.

**Case presentation:**

We present this case to describe a concomitant medical and surgical management of a living 11 + 5 weeks cervical pregnancy. The initial beta-human chorionic gonadotropins (ß-hCG) serum level was 108,730 IU/L. The patient received 60 mg of methotrexate intra-amniotically followed by another dose of 60 mg of methotrexate intramuscularly 24 h later. Fetal heartbeats stopped on day 03. On day 07, the ß-hCG was 37,397 IU/L. On day 13, the patient had evacuation of the remaining products of conception with the insertion of an intracervical Foley catheter to minimize the bleeding. On day 34, the ß-hCG was negative.

**Conclusion:**

The concomitant use of methotrexate to induce fetal demise along with surgical evacuation may be considered in the management of advanced cervical pregnancy to avoid excessive blood loss, and ultimately hysterectomy.

## Background

Cervical pregnancy is a rare type of ectopic pregnancy. The estimated prevalence is one in 10,000 deliveries and 1% of all ectopic pregnancies [1]. The management of cervical pregnancy is challenging because of the rarity of the condition, late presentation, which is associated with increased risk of failed medical treatment, and excessive post-evacuation bleeding that may require hysterectomy. Methotrexate therapy was reported to have higher chances of success in cervical pregnancies less than 12 weeks of gestation [2, 3]. A retrospective study of 62 cases reported that gestational age of more than 9 + 0 weeks, ß-hCG level of more than 10,000 IU/l, crown-rump length of more than 10 mm, and fetal cardiac activity were associated with a higher risk of failure of systemic methotrexate therapy [4]. Additionally, the authors reported that combination therapy of systemic and intra-amniotic injection of methotrexate seemed to increase the chance of successful treatment [4]. Moreover, there is no good evidence in the literature regarding the pharmacological management of living cervical ectopic pregnancy of more than 9 + 0 weeks of gestation, and there is no standard protocol on methotrexate doses in these cases. We present this case to describe a concomitant medical and surgical management of a relatively advanced living cervical pregnancy, which bears the risk of failure of methotrexate therapy, and the risk of severe uncontrolled bleeding if surgical evacuation was attempted alone.

## Case presentation

**Fig. 1:**
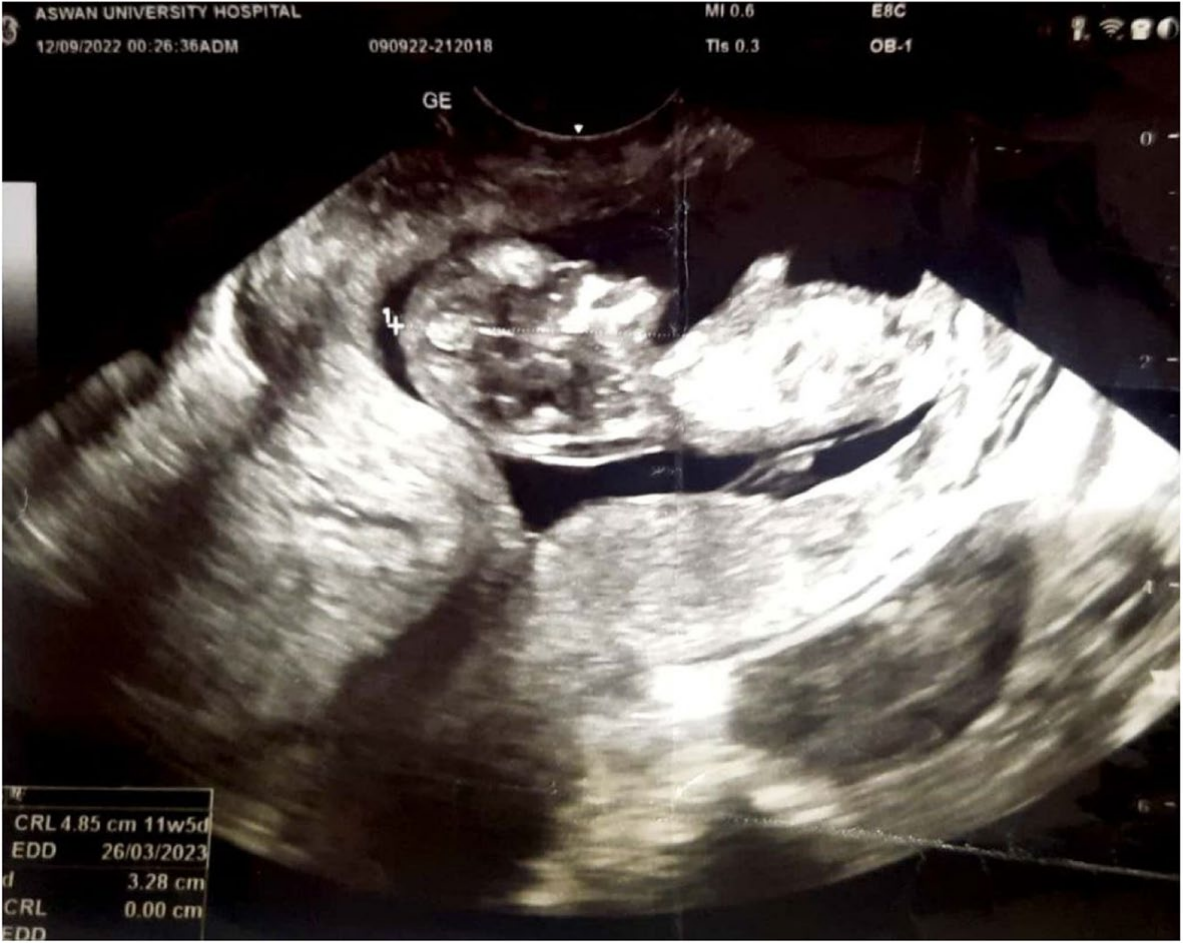
Transvaginal ultrasound on day 0 showing cervical ectopic pregnancy with crown-rump length measuring 48.5 mm, corresponding to 11 + 5 weeks

A 27-year-old primigravida patient, at 10 + 1 weeks of amenorrhea, with a spontaneously conceived pregnancy, was referred to the obstetrics and gynecology department, Aswan University Hospitals for assessment of the fetal location. The patient did not complain of any abdominal pain or abnormal vaginal bleeding. The vital signs of the patient were stable. The ultrasound machine used was Voluson S8, with a transvaginal ultrasound probe of 7 MHz (Voluson™, GE Healthcare Technologies Inc., Chicago, Illinois, USA). The transvaginal ultrasound revealed an empty uterus, and the gestational sac was seen occupying the cervix with a characteristic barrel-shaped sign (Fig. 1). The crown-rump length was 48.5 mm, corresponding to 11 + 5 weeks. The fetal heart rate was 160 beats/minute, and the fetal movements were appreciated at the time of the ultrasound examination. The placenta was seen at the caudal part of the cervix. A color Doppler ultrasound confirmed the placental location and showed a retroplacental line of high blood velocity. When applying gentle pressure using the transvaginal ultrasound probe, the gestational sac was not sliding against the cervical canal (negative sliding sign). There was no free fluid in the pouch of Douglas, and both adnexa were looking normal. The ß-human chorionic gonadotropin (ß-hCG) serum level was 108,730 IU/L on the day of presentation. The patient’s height was 1.6 m, and her weight was 60 kg. The patient was admitted to the inpatient ward and was counseled by two senior obstetricians about the administration of systemic plus intraamniotic methotrexate therapy.

The calculated dose of methotrexate was 60 mg (1 mg/kg). The plan was to inject 60 mg of methotrexate intra-amniotically via a suprapubic insertion of a spinal anesthesia cannula and to administer another dose of 60 mg of methotrexate intramuscularly after 24 h. The suprapubic route was agreed on rather than the transcervical route to avoid the transplacental insertion of the cannula and the resultant significant bleeding. An informed written consent was obtained from the patient after explaining the benefits and the risks of the procedure.

The day of the procedure (day 0) was on the day after the admission. The patient was transferred to the theater room and spinal anesthesia was administrated. After the effect of spinal anesthesia ensued, evacuation of the bladder was done using a metal urinary catheter, and the patient was put in a lithotomy position. A bimanual examination of the patient was done, and the cervical pregnancy could be palpated two-finger breadth above the symphysis pubis. Under the transvaginal ultrasound guidance, we inserted a 20-gauge spinal anesthesia cannula in the suprapubic region directed towards the cervical pregnancy to administer the intraamniotic methotrexate. Once the cannula was seen inside the amniotic cavity, 2 cm of amniotic fluid was aspirated to confirm the location of the cannula. Then 60 mg of methotrexate was injected slowly inside the amniotic cavity. The flush of the methotrexate solution was appreciated on the ultrasound machine.

The patient was transferred back to the inpatient ward. The pulse and blood pressure were closely monitored in the first 2 h, and they remained within the normal values. After 12 h, a transabdominal ultrasound showed that the fetal heartbeats were still visible at 150 beats/minute. There was no free fluid in the pouch of Douglas. The patient did not report any pain or vaginal bleeding on the day of the procedure.

On the day after the procedure (day 01), 60 mg of methotrexate was administered intramuscularly. On day 02, the patient reported minimal vaginal spotting; however, the fetal heartbeats were still visible at 155 beats/minute, but the fetal movements were significantly diminished. On day 03, a color Doppler ultrasound confirmed the absence of fetal cardiac activity, and the pregnancy was anhydramnios.

On day 07 after the procedure, the ß-hCG serum level was 37,397 IU/L. On day 13, the cervical pregnancy completely collapsed on ultrasound. We counseled the patient about suction and evacuation of the products of conception to expedite her recovery and shorten the duration of her hospital stay. The patient was transferred to the theatre on day 13 for suction evacuation of the products of conception. Mild bleeding was noted from the pregnancy site during the procedure, and an intracervical Foley catheter balloon was compressed against the pregnancy site and successfully controlled the bleeding. The patient’s pulse and blood pressure were within normal levels in the first 24 h after the suction evacuation procedure.

On day 15 after the procedure, the ß-hCG serum level was 239. The patient was then discharged and was asked to perform a serum ß-hCG level weekly. On day 34, the ß-hCG was negative (Table 1).

**Table 1:** Follow-up of the ß-human chorionic gonadotropins after methotrexate injection

Day	ß-hCG (IU/L)
At presentation	108,730
Day 07 after the methotrexate injection	37,397
Day 15 after the methotrexate injection	239
Day 34 after the methotrexate injection	Negative

## Discussion and conclusion

We present a concomitant medical and surgical management of a living cervical pregnancy at 10 weeks of gestation. The case was a primigravida conceived via spontaneous pregnancy with no known risk factors for cervical pregnancy. The relatively advanced gestation age, the high initial ß-hCG, and the viability of the fetus were risk factors for failure of systemic methotrexate therapy. Additionally, the advanced gestational age with the caudal location of the placenta would result in serious hemorrhage during the surgical evacuation that may necessitate hysterectomy. In this case, we induced fetal demise with intraamniotic and systemic methotrexate with later surgical evacuation after the collapse of the gestational sac. The amount of bleeding noticed during the evacuation was mild and it was successfully controlled by compression of an intracervical Foley catheter balloon.

Cervical pregnancy is a rare condition; for this reason, most of the published articles are case reports. There are many lines of management described in the literature, including local methotrexate therapy, systemic methotrexate therapy, curettage, uterine artery embolization, and a combination of multiple lines. Hysterectomy is reserved for cases with uncontrolled bleeding after a trial of curettage or after spontaneous rupture of cervical pregnancy. Due to the lack of comparative studies, there is no consensus on the criteria for medical, surgical, or concomitant medical and surgical treatment of cervical ectopic pregnancy.

Initial management with systemic methotrexate therapy is one of the initial lines of management of early cervical pregnancy. In a retrospective study by Vela et al., they reported 12 cases of cervical pregnancy from a single center between 1985 and 2005. A single dose of 50 mg/m2 methotrexate was administrated to four patients; only one patient, with ß-hCG of 4,029 mIU/ml with fetal cardiac activity, had complete resolution with no further management. The other three cases required additional treatment with a second dose of methotrexate, uterine artery embolization, or curettage. The ß-hCG levels of these three cases were 22,324 mIU/ml, 6,107 mIU/ml, and 380 mIU/ml, and the fetal cardiac activity was present [1]. Similarly, a single-center study reported by Stabile et al., between 2014 to 2020, described six cases of cervical pregnancy. Only one case was managed medically using a combination of a single dose of systemic methotrexate (50 mg/m2) with oral mifepristone and misoprostol. In this case, the GSD was 4.7 × 5 with an anembryonic pregnancy, and the ß-hCG level was 1,331 mIU/ml. Based on these reported cases, the regimen of a single dose of systemic methotrexate therapy has limitations in the presence of fetal cardiac activity in the management of cervical pregnancy [5].

In the literature, late diagnosis, high initial ß-hCG, and fetal viability are associated with a higher failure rate of methotrexate therapy. A Review article conducted by Hung et al., which included 48 cases from 28 publications, concluded that the main criteria for methotrexate therapy were serum ß-hCG ≥ 10,000 mIU/ml (Odds ratio (OR) = 10.8%, 95% confidence interval (CI) = 2.6–45.1), crown-rump length of > 10 mm (OR = 13.33, 95% CI = 1.46, 120.48), visualization of fetal heart beats (OR = 14.29, 95% CI = 2.95–76.92), and gestational age at presentation ≥ 9 weeks (OR = 6.44, 95% CI = 1.46–28.52) [6]. Similarly, a retrospective study by Kung et al. reported 62 cases of living and non-living cervical pregnancies. They concluded that living cervical pregnancies (43%) are more likely to require a concomitant surgical intervention when compared to non-living ones (13%) with a statistically significant difference (*p* = 0.021) [4].

The administration of a feticide agent was shown to improve the effect of systemic methotrexate in several case reports. In the study of Hung et al., feticide using either intraamniotic KCL, intraamniotic methotrexate, or an ultrasound-guided puncture of the fetal heart was found to improve the therapeutic effect of methotrexate treatment (OR = 0.13, 95% CI = 0.02–0.68). However, higher doses of methotrexate (> 150 mg total dose) did not seem to be more effective than lower doses (adjusted OR = 1.05, *p* = 0.97) [6]. A case report by Ozcivit et al. described a combined intraamniotic and systemic methotrexate therapy in a living 11-week cervical pregnancy with an initial ß-hCG of 10,000 mIU/ml. The total dose they used was 75 mg of methotrexate, two-thirds of this dose was injected intra-amniotically, and the remaining third was administrated systemically. The case was completely resolved without the need for any further management [7].

Concomitant surgical and medical treatment has been described in the literature. In the retrospective study by Stabile et al., they described two cases managed with systemic methotrexate followed by hysteroscopic removal of the conceptus with the use of electrosurgery to control the bleeding. The first case had an initial ß-hCG of 4,272 mIU/ml, and the gestational sac diameter (GSD) was 5 × 5.3 mm; however, they did not report the status of the fetal cardiac activity. The second case was relatively advanced with ß-hCG of 9,747, and GSD of 20 × 12 mm. Both cases had a complete resolution. They suggested that the hysteroscopic approach with or without methotrexate is safer than traditional methods of curettage, as it offers direct visualization and precise cauterization of the bleeding vessels [5].

Surgical management of cervical pregnancy, in the form of suction evacuation or curettage, carries a risk for serious hemorrhage that may require an emergency hysterectomy. Among the six cases described by Stabile et al., one case had hysterectomy with uterine artery embolization. The described case was 12 + 1 weeks of gestation with an embryonic gestational sac measuring 78 × 60 mm, and the ß-hCG was 97,388 mIU/ml [5]. Additionally, in Vela et al. study, five cases of cervical pregnancy out of 12 had hysterectomy. The indication of hysterectomy was uncontrolled bleeding after curettage in four cases and spontaneous rupture of cervical pregnancy in the fifth case [1].

In conclusion, the concomitant use of methotrexate to induce fetal demise along with surgical evacuation may be considered in the management of advanced cervical pregnancy to avoid excessive blood loss and ultimately hysterectomy.

## Data Availability

The authors declare that the data pertinent to this case report is available upon request.

## Abbreviation

ß-hCG: ßeta human chorionic gonadotropin
